# Remnant Cholesterol to Lymphocyte Ratio as a New Predictor of Prognosis in Patients with Unstable Angina Undergoing Percutaneous Coronary Intervention

**DOI:** 10.31083/j.rcm2403071

**Published:** 2023-02-28

**Authors:** Hui Xi, Biyang Zhang, Tienan Sun, Jingrui Zhang, Haichen Lv

**Affiliations:** ^1^Peking University International Hospital, 102218 Beijing, China; ^2^Beijing Anzhen Hospital affiliated Capital Medical University, 100089 Beijing, China; ^3^Cardiovascular Hospital of Dalian Medical University, The First Affiliated Hospital of Dalian Medical University, 116000 Dalian, Liaoning, China

**Keywords:** remnant cholesterol, remnant cholesterol-lymphocyte ratio, unstable angina, percutaneous coronary intervention, major adverse cardiovascular event

## Abstract

**Background:**

Inflammatory cells and remnant cholesterol (RC) play an important role in the development and progression of cardiovascular diseases. In order to understand their contribution to cardiovascular diseases, we proposed the RC to lymphocyte ratio (RCLR) that reflects the level of serum lipid and inflammation as a predictive indicator. In this study, we explored the correlation between RCLR and major adverse cardiovascular events (MACEs) in patients with unstable angina (UA) treated with percutaneous coronary intervention (PCI).

**Methods:**

RCLR was calculated by dividing RC by lymphocyte percentage. Patients were divided into four groups according to RCLR quartiles. The endpoint of the study was MACE, a composite endpoint including all-cause mortality, non-fatal myocardial infarction (MI), and ischemia‑driven revascularization. The multivariable Cox proportional hazard model was used to determine the exclusive effect of RCLR on MACE.

**Results:**

The study was conducted on 1092 patients with UA. The rate of MACE increased as RCLR quartiles increased (quartile 4 vs quartile 1: 40.9% vs 9.2%, *p *< 0.001). An adjustment for confounding variables revealed that an increase in the rate of MACE was directly proportional to RCLR (quartile 4 vs quartile 1: HR - 5.85 [95% CI, 3.77–9.08], *p *< 0.001, *p* for trend < 0.001).

**Conclusions:**

RCLR independently correlated with the incidence of MACE in patients with UA treated with PCI.

## 1. Introduction

Despite being treated with percutaneous coronary intervention (PCI), coronary artery disease (CAD) remains the leading cause of morbidity and mortality worldwide [1, 2]. Dyslipidemia is an established risk factor for CAD. Consequently, serum lipids have been studied as key therapeutic targets for CAD in the past few decades [3, 4, 5, 6]. Different kinds of studies, including observational and genetic studies as well as randomized controlled trials, have shown that an increase in low-density lipoprotein cholesterol (LDL-C) leads to a higher risk of adverse cardiovascular outcomes [7]. However, there is still considerable residual cardiovascular risk after LDL-C reaches the levels the guidelines recommended [8]. Studies in recent years have shown that remnant cholesterol (RC) might be responsible for this residual risk [9]. Previous studies have revealed a significant association between RC and adverse cardiovascular consequences [10, 11, 12].

On the other hand, inflammation plays an important role in the development of CAD [13, 14, 15]. The lymphocyte count in peripheral blood can reflect the inflammatory state of the body. Moreover, previous reports have suggested that a reduction in lymphocyte count is associated with adverse outcomes in cardiovascular diseases [16, 17, 18, 19].

In the present study, we hypothesized that the RC to lymphocyte ratio (RCLR), an index for the combination of lipide and inflammation, might be associated with adverse cardiovascular events in patients with unstable angina (UA), which is one of the important components of CAD. Hence, we conducted a retrospective study to examine the ability of RCLR in predicting the incidence of major adverse cardiovascular events (MACE) among UA patients treated with PCI.

## 2. Method

### 2.1 Study Population

Patients with UA hospitalized for PCI at Beijing Anzhen Hospital from January 2015 to December 2015 were enrolled in this study. The study exclusion criteria were as follows: (1) age <18 years; (2) body mass index (BMI) >45 kg/$m^{2}$; (3) suspected familial hypertriglyceridemia (triglyceride ≥5.65 mmol/L); (4) left ventricular ejection fraction (LVEF) <30%; (5) receiving chemotherapy due to cancer (including hematologic malignancy); (6) missing data on total cholesterol, high-density lipoprotein cholesterol (HDL-C), and LDL-C; (7) patients with missing personal data ≥5%; (8) history of coronary artery bypass grafting; (9) rheumatic diseases. Patients included in this study were then divided into four groups, as per the RCLR quartiles.

### 2.2 Data Extraction

The following data were recorded in this study: demographics (age, gender), vital signs (blood pressure and heart rate), BMI, smoking and drinking habits, medical history (hypertension, diabetes, prior myocardial infarction [MI], PCI, stroke); cholesterol-lowering therapy (statins or ezetimibe); laboratory parameters (white blood cell [WBC], alanine aminotransferase [ALT], aspartate aminotransferase [AST], lymphocyte and neutrophil percentage, hemoglobin, platelet, creatinine, estimated glomerular filtration rate, triglycerides, total cholesterol, HDL-C, LDL-C, RC, sodium, potassium, LVEF); coronary angiography results (left main coronary artery [LM] disease, triple-vessel disease, chronic total occlusion [CTO], diffuse lesion, bifurcation lesion); target vessel, and number of stents.

RC was estimated as total cholesterol minus LDL-C minus HDL-C, and RCLR was calculated by dividing RC by lymphocyte percentage.

### 2.3 Outcomes and Follow-Up

The primary outcome was the composite endpoint, MACE, including all-cause mortality, non-fatal MI, and ischemia-driven revascularization. All patients were followed up annually for up to five years by trained professionals after PCI treatment. Relevant information about the poor prognostic outcome was obtained from patients or their families through a telephone questionnaire.

### 2.4. Statistical Analysis

Normally distributed continuous variables were expressed as mean ± standard deviation and compared between groups using analysis of variance. Non-normally distributed data were expressed as median and the interquartile range and compared using the Kruskal–Wallis test. Categorical variables were expressed as numbers (percentages) and compared between groups using the Chi-square test.

The 5-year incidence of adverse events in each group was described by Kaplan-Meier curves, and the log-rank test was used to compare the differences between the groups. Multiple Cox regression analyses were performed to further investigate the independent association of adverse events. The group of the first RCLR quartile served as the reference group, and the results of multiple Cox regression analyses were summarized as hazard ratio (HR) and 95% confidence interval (CI). *p*-value for trend was calculated. Covariables included in the model were selected according to statistical analysis and clinical suspicion. The forest map was drawn to visually demonstrate the influence of each variable on MACE. Receiver operating characteristic (ROC) analysis was applied to assess the ability of RCLR and RC in predicting the incidence of non-fatal MI, ischemia-driven revascularization, and MACE; all-cause mortality was not included because of low incidence. Differences between the area under the ROC curve (AUC) of the two indices were compared using the DeLong test. All tests were two-sided, and statistical significance was set at *p *< 0.05. All methods were performed in accordance with relevant guidelines and regulations. All data analyses were performed using Stata (v.15.1, 4905 Lakeway Drive, CS, Texas, USA).

## 3. Result

### 3.1 Subjects and Baseline Characteristics

The study was conducted on 1092 patients with UA. All patients were stratified into four groups according to RCLR quartiles: RCLR <1.58 (n = 272), 1.58 ≤ RCLR < 2.21 (n = 275), 2.21 ≤ RCLR < 3.07 (n = 271), RCLR ≥3.07 (n = 274). Patients with higher RCLR tended to be younger and have a higher BMI as well as heart rate. In terms of laboratory parameters, patients with higher RCLR had higher WBC count, ALT, AST, neutrophil percentage, triglyceride, total cholesterol, LDL-C, and RC, while their lymphocyte percentage, platelet, HDL-C, and sodium levels were lower. Patients in the higher RCLR quartile groups were more likely to have triple-vessel lesions, as detected by coronary angiography. Moreover, 99.3% of all patients received cholesterol-lowering therapy. Specifically, 98.9% received statins and 16.9% received ezetimibe. No statistical differences in cholesterol-lowering therapy were observed between the groups (Table 1).

**Table 1.** **Characteristics**.

		Total	RCLR <1.58	1.58 ≤ RCLR < 2.21	2.21 ≤ RCLR < 3.07	RCLR ≥3.07	*p*
		(n = 1092)	(n = 272)	(n = 275)	(n = 271)	(n = 274)	
Age, year		65.3 ± 9.8	65.9 ± 9.7	65.9 ± 9.5	65.6 ± 10.0	63.9 ± 10.0	0.044
Gender (male), n (%)		790 (72.3)	203 (74.6)	200 (72.7)	196 (72.3)	191 (69.7)	0.641
BMI, kg/$m^{2}$		25.5 ± 3.3	25.2 ± 3.0	25.3 ± 3.6	25.7 ± 3.2	26.0 ± 3.2	0.020
Heartrate, beat/min		70.2 ± 10.0	68.6 ± 8.7	70.2 ± 9.2	70.1 ± 10.4	71.7 ± 11.1	0.004
SBP, mmHg		134.6 ± 17.0	133.9 ± 18.3	135.3 ± 16.5	134.9 ± 15.7	134.3 ± 17.3	0.793
DBP, mmHg		79.6 ± 9.9	79.0 ± 9.7	79.6 ± 9.9	79.8 ± 9.8	80.0 ± 10.5	0.716
Smoker, n (%)		627 (57.4)	160 (58.8)	161 (58.6)	162 (59.8)	144 (52.6)	0.304
Drinker, n (%)		264 (24.2)	60 (22.1)	76 (27.6)	65 (24.0)	63 (23.0)	0.444
Complication, n (%)							
	Hypertension	682 (62.5)	157 (57.7)	166 (60.4)	175 (64.6)	184 (67.2)	0.102
	Diabetes mellitus	348 (31.9)	83 (30.5)	79 (28.7)	94 (34.7)	92 (33.6)	0.417
	Prior MI	190 (17.4)	50 (18.4)	45 (16.4)	46 (17.0)	49 (17.9)	0.926
	Prior PCI	105 (9.6)	28 (10.3)	26 (9.5)	21 (7.8)	30 (11.0)	0.615
	Prior stroke	145 (13.3)	44 (16.2)	34 (12.4)	35 (12.9)	32 (11.7)	0.420
Cholesterol lowering therapy		1084 (99.3)	271 (99.6)	274 (99.6)	268 (98.9)	271 (98.9)	0.566
	Statins	1080 (98.9)	269 (98.9)	274 (99.6)	266 (98.2)	271 (98.9)	0.431
	Ezetimibe	185 (16.9)	48 (17.7)	48 (17.5)	46 (17.0)	43 (15.7)	0.929
Laboratory parameters							
	WBC, ${\mathrm{10}}^{9}$/L	7.3 ± 1.4	6.9 ± 1.7	7.1 ± 1.5	7.2 ± 1.6	7.9 ± 2.0	<0.001
	Neutrophil percentage, %	63.6 ± 8.4	59.4 ± 7.9	62.5 ± 7.1	64.3 ± 7.7	68.3 ± 8.5	<0.001
	Lymphocyte percentage, %	28.5 ± 7.7	32.5 ± 7.4	29.5 ± 6.1	28.0 ± 7.2	24.0 ± 7.6	<0.001
	Platelet, ${\mathrm{10}}^{9}$/L	225.9 ± 55.8	218.7 ± 57.1	226.9 ± 53.6	224.3 ± 52.9	223.6 ± 58.5	0.017
	Hemoglobin, g/L	140.5 ± 14.1	138.9 ± 13.9	140.8 ± 14.3	140.6 ± 13.5	141.6 ± 14.5	0.142
	ALT, U/L	32.2 ± 22.9	29.3 ± 21.7	31.2 ± 20.9	35.7 ± 26.9	32.5 ± 21.5	0.011
	AST, U/L	26.5 ± 15.4	24.9 ± 12.5	25.8 ± 13.8	28.6 ± 18.5	26.7 ± 15.9	0.035
	Creatinine, μmol/L	75.6 ± 16.3	74.8 ± 16.4	74.4 ± 15.1	77.5 ± 17.4	75.7 ± 16.2	0.121
	eGFR, mL/min*1.73 $m^{2}$	94.2 ± 20.5	95.8 ± 20.4	95.5 ± 20.6	91.7 ± 20.5	93.7 ± 20.2	0.077
	Triglyceride, mmol/L	2.0 ± 1.3	1.3 ± 0.4	1.6 ± 0.5	2.1 ± 0.7	3.2 ± 1.9	<0.001
	Total cholesterol, mmol/L	4.2 ± 1.1	3.8 ± 0.9	4.0 ± 0.9	4.2 ± 1.0	4.7 ± 1.2	<0.001
	HDL-C, mmol/L	1.0 ± 0.2	1.0 ± 0.2	1.0 ± 0.2	1.0 ± 0.2	0.9 ± 0.2	<0.001
	LDL-C, mmol/L	2.5 ± 0.9	2.4 ± 0.8	2.4 ± 0.8	2.5 ± 0.9	2.6 ± 1.0	0.002
	RC, mmol/L	0.7 ± 0.5	0.4 ± 0.1	0.6 ± 0.1	0.7 ± 0.2	1.2 ± 0.6	<0.001
	Sodium, mmol/L	140.1 ± 2.2	140.6 ± 2.3	140.3 ± 2.1	140.1 ± 2.1	139.4 ± 2.3	<0.001
	Potassium, mmol/L	4.3 ± 0.3	4.3 ± 0.3	4.3 ± 0.3	4.3 ± 0.3	4.3 ± 0.3	0.432
	LVEF, %	66.2 ± 6.5	66.0 ± 7.2	66.2 ± 7.5	66.3 ± 6.1	66.4 ± 6.4	0.926
Coronary angiography results, n (%)							
	LM disease	48 (4.4)	11 (4.0)	10 (3.6)	12 (4.4)	15 (5.5)	0.749
	Triple-vessel disease	304 (27.8)	76 (27.9)	73 (26.6)	62 (22.9)	93 (33.9)	0.035
	CTO	48 (4.4)	16 (5.9)	13 (4.7)	8 (3.0)	11 (4.0)	0.401
	Diffuse lesion	312 (28.6)	67 (24.6)	80 (29.1)	77 (28.4)	88 (32.1)	0.284
	Bifurcation lesion	244 (22.3)	51 (18.8)	66 (24.0)	68 (25.1)	59 (21.5)	0.291
Target vessel territory, n (%)							
	LM	47 (4.3)	13 (4.8)	10 (3.6)	12 (4.4)	12 (4.4)	0.927
	LAD	882 (80.8)	224 (82.4)	225 (81.8)	222 (81.9)	211 (77.0)	0.339
	LCX	553 (50.6)	125 (45.9)	134 (48.7)	134 (49.5)	160 (58.4)	0.023
	RCA	619 (56.7)	152 (55.9)	156 (56.7)	140 (51.7)	171 (62.4)	0.089
Number of stents		2 (1–3)	2 (1–2)	2 (1–3)	2 (1–3)	2 (1–3)	0.089

Abbreviation: BMI, body mass index; SBP, systolic blood pressure; DBP, diastolic blood pressure; MI, myocardial infarction; PCI, percutaneous coronary intervention; WBC, white blood cell; ALT, alanine aminotransferase; AST, aspartate aminotransferase; eGFR, estimated glomerular filtration rate; HDL-C, high-density lipoprotein cholesterol; LDL-C, low-density lipoprotein cholesterol; RC, remnant cholesterol; LVEF, left ventricular ejection fraction; LM, left main coronary artery; CTO, chronic total occlusion; LAD, left anterior descending artery; LCX, left circumflex artery; RCA, right coronary artery; RCLR, remnant cholesterol to lymphocyte ratio.

### 3.2 Association between RCLR and MACE

The overall all-cause mortality rate was 2.5%. Patients in the highest RCLR quartile did not show a significant increase in mortality compared to those in the lowest quartile (quartile 4 vs. quartile 1: 4.0% vs. 1.1%, *p* = 0.177). The rates of non-fatal MI and ischemia-driven revascularization were 8.7% and 12.2%, respectively. These rates increased significantly in higher RCLR quartiles (non-fatal MI: quartile 4 vs. quartile 1: 16.1% vs. 3.7%, *p *< 0.001; ischemia-driven revascularization: quartile 4 vs. quartile 1: 20.8% vs. 4.4%, *p *< 0.001). The overall MACE rate was 23.4% and a higher RCLR was associated with an increased MACE rate (quartile 4 vs. quartile 1: 40.9% vs. 9.2%, *p *< 0.001) (Table 2).

**Table 2.** **Adverse events**.

	Total	RCLR <1.58	1.58 ≤ RCLR < 2.21	2.21 ≤ RCLR < 3.07	RCLR ≥3.07	*p*
	(n = 1092)	(n = 272)	(n = 275)	(n = 271)	(n = 274)	
All-cause death, n (%)	27 (2.5)	3 (1.1)	6 (2.2)	7 (2.6)	11 (4.0)	0.177
Non-fatal MI, n (%)	95 (8.7)	10 (3.7)	17 (6.2)	24 (8.9)	44 (16.1)	<0.001
Ischemia‑driven revascularization, n (%)	133 (12.2)	12 (4.4)	29 (10.6)	35 (12.9)	57 (20.8)	<0.001
MACE, n (%)	255 (23.4)	25 (9.2)	52 (18.9)	66 (24.4)	112 (40.9)	<0.001

Abbreviation: RCLR, remnant cholesterol to lymphocyte ratio; MACE, major adverse cardiovascular.

**Fig. 1.**
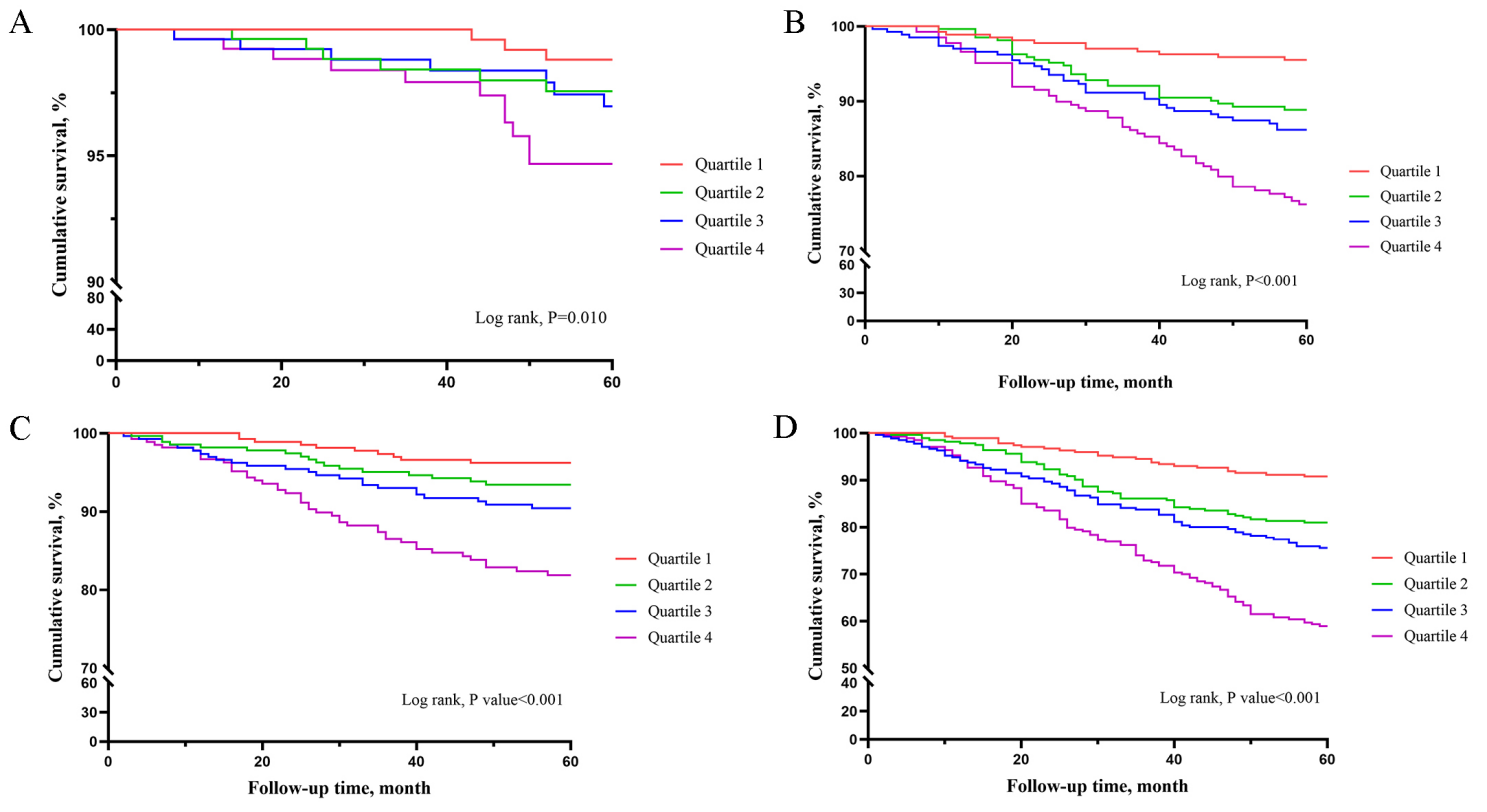
**Kaplan-Meier curves showing the association between RCLR quartiles and clinical outcomes**. (A) Kaplan-Meier curves showing the association between RCLR quartiles and death. (B) Kaplan-Meier curves showing the association between RCLR quartiles and ischemia-driven revascularization. (C) Kaplan-Meier curves showing the association between RCLR quartiles and non-fatal MI. (D) Kaplan-Meier curves showing the association between RCLR quartiles and MACE.

**Fig. 2.**
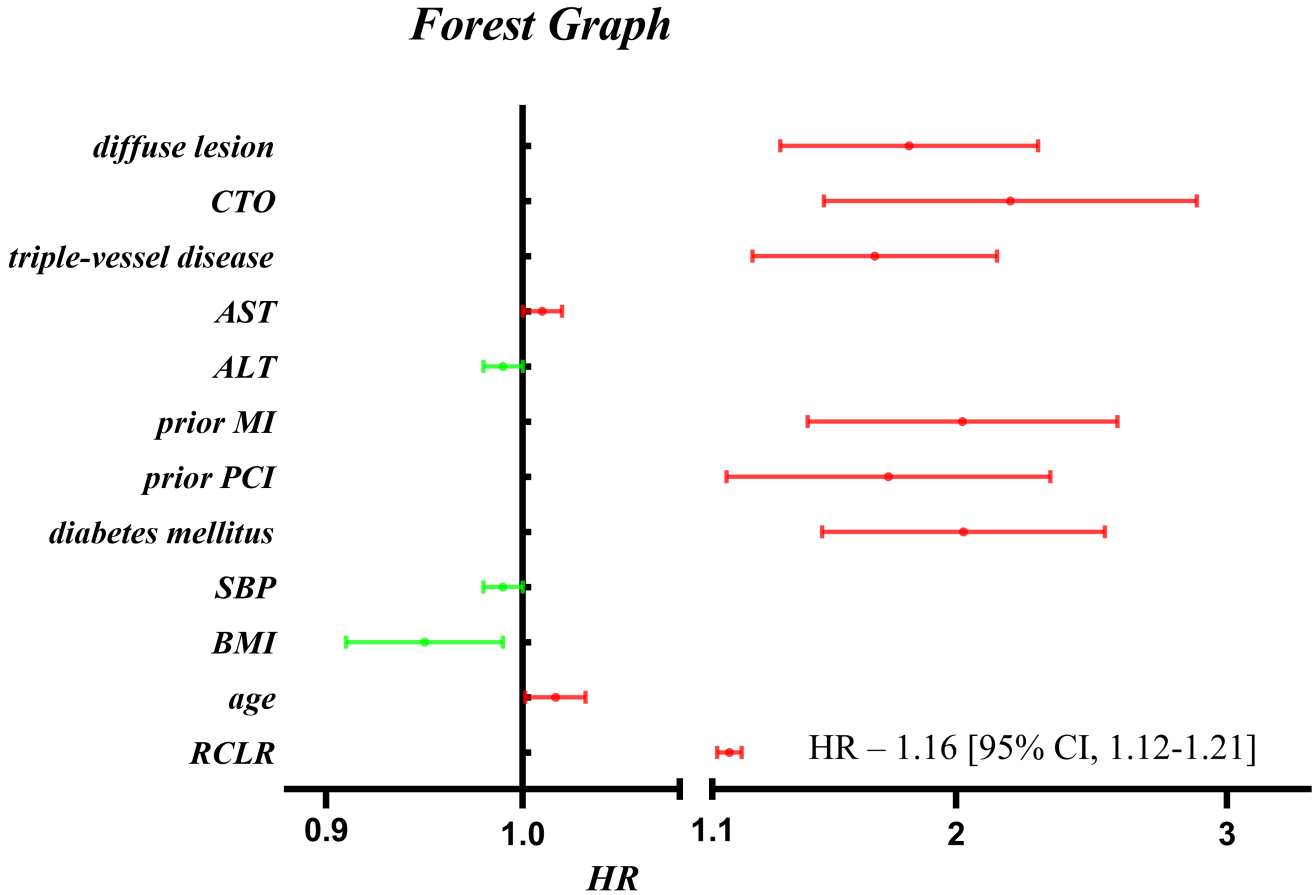
**The forest map demonstrating the influence of each variable on MACE in multivariable Cox proportional hazards model**.

The Kaplan-Meier curves (Fig. 1) revealed that higher RCLR quartiles were associated with higher incidence of all-cause mortality (log-rank, *p* = 0.010), non-fatal MI (log-rank, *p *< 0.001), ischemia-driven revascularization (log-rank, *p *< 0.001), and MACE (log-rank, *p *< 0.001). The direct effect of RCLR on adverse events was confirmed by Cox regression analysis. After the adjustment for potential confounding variables, such as age, AST, ALT, BMI, systolic blood pressure (SBP), CTO, diffuse lesion, prior MI, triple-vessel disease, prior PCI, and diabetes mellitus, included in the final model, a positive correlation was noted between the RCLR and mortality (quartile 4 vs. quartile 1: HR - 5.42 [95% CI, 1.47–20.00], *p* = 0.011, *p* for trend < 0.001). Higher RCLR quartiles were associated with an increased risk of non-fatal MI (quartile 4 vs. quartile 1: HR - 6.74 [95% CI, 3.34–13.59], *p *< 0.001, *p* for trend < 0.001) and ischemia-driven revascularization (quartile 4 vs. quartile 1: HR - 5.47 [95% CI, 2.92–10.25], *p *< 0.001, *p* for trend < 0.001). Moreover, RCLR was directly related to an increased risk of MACE (quartile 4 vs. quartile 1: HR - 5.85 [95% CI, 3.77–9.08], *p *< 0.001, *p* for trend <0.001). The forest map was drawn to visually demonstrate the influence of each variable on MACE (RCLR was considered as a continuous variable) (Fig. 2). According to Fig. 2, we found that indicators such as RCLR (HR - 1.16 [95% CI, 1.12–1.21], *p *< 0.001), age, AST, prior MI, prior PCI, diabetes mellitus, triple-vessel disease, CTO, and diffuse lesion significantly increased the incidence of MACE, while others decreased the incidence of MACE markedly. When RCLR was considered as a continuous variable in the model for analysis, we observed that RCLR levels were associated with a 0.16-, 0.18-, and 0.16-fold increase in the risk of non-fatal MI, ischemia-driven revascularization, and MACE, respectively. However, we could not find a statistically significant association between RCLR and all-cause death (Table 3).

**Table 3.** **The effect of RCLR on clinical outcomes by Cox regression analysis**.

		Multivariable Cox proportional hazards model		
		HR (95% CIs)	*p*	*p* for trend
All-cause death				<0.001
	Quartile 1	1.0 (Ref)		
	Quartile 2	2.13 (0.52–8.67)	0.292	
	Quartile 3	2.68 (0.68–10.59)	0.159	
	Quartile 4	5.42 (1.47–20.00)	0.011	
	Continuous variable	1.13 (0.97–1.32)	0.127	
Non‑fatal MI				<0.001
	Quartile 1	1.0 (Ref)		
	Quartile 2	2.01 (0.91–4.42)	0.083	
	Quartile 3	3.21 (1.51–6.80)	0.002	
	Quartile 4	6.74 (3.34–13.59)	<0.001	
	Continuous variable	1.16 (1.09–1.23)	<0.001	
Ischemia-driven revascularization				<0.001
	Quartile 1	1.0 (Ref)		
	Quartile 2	2.45 (1.24–4.82)	0.010	
	Quartile 3	3.22 (1.66–6.25)	0.001	
	Quartile 4	5.47 (2.92–10.25)	<0.001	
	Continuous variable	1.18 (1.12–1.24)	<0.001	
MACE				<0.001
	Quartile 1	1.0 (Ref)		
	Quartile 2	2.26 (1.40–3.65)	0.001	
	Quartile 3	3.15 (1.98–5.02)	<0.001	
	Quartile 4	5.85 (3.77–9.08)	<0.001	
	Continuous variable	1.16 (1.12–1.21)	<0.001	

Models were derived from multivariate Cox regression analysis. Multivariable Cox proportional hazards model: adjusted for age, AST, ALT, BMI, SBP, CTO, diffuse lesion, prior MI, triple-vessel disease, prior PCI, diabetes mellitus. Abbreviation: OR, odds ratio; CI, confidence interval.

**Fig. 3.**
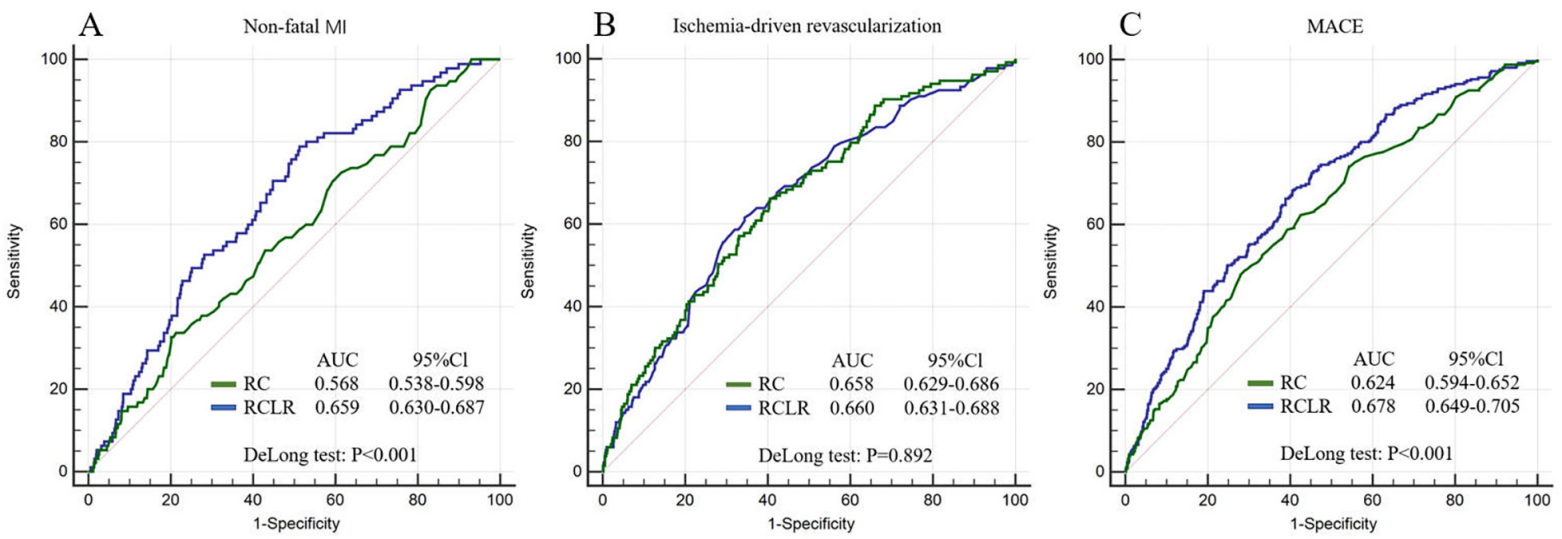
**ROC curves for adverse outcomes**. (A) ROC curves of RCLR and RC for non-fatal MI. (B) ROC curves of RCLR and RC for ischemia-driven revascularization. (C) ROC curves of RCLR and RC for MACE.

The ability to predict the primary endpoints of RCLR is presented in Fig. 3. The AUCs of RCLR for a 5-year incidence of non-fatal MI, ischemia-driven revascularization, and MACE were 0.659, 0.660, and 0.678, respectively. The ability of RCLR to predict non-fatal MI and MACE was significantly higher than that of RC (RCLR vs. RC for predicting both, *p *< 0.001). However, there was no statistically significant difference between RCLR and RC for the prediction of ischemia-driven revascularization (*p* = 0.892).

## 4. Discussion

Our study demonstrated an association between RCLR and MACE in UA patients treated with PCI. The major findings of our study are as follows. (1) In the higher RCLR quartiles, the incidence of MACE was significantly higher. After the adjustment of possible confounding variables, a higher RCLR was found to be directly associated with higher incidence of MACE. Additionally, our results reveal a strong association of RCLR with all-cause death, non-fatal MI, and ischemia-driven revascularization by multiple Cox regression analysis. (2) The Kaplan-Meier curves also showed that higher RCLR quartiles were associated with higher incidence of MACE. (3) In terms of efficiency, RCLR was found to be capable of predicting the incidence of MACE much better than RC in UA patients treated with PCI.

The connection between a higher RCLR and increased rate of MACE is biologically plausible. RC is a component of triglyceride-rich lipoproteins (TRLs). TRLs are able to cross the artery wall, after which they are absorbed by macrophages and smooth muscle cells. During the metabolism of TRL inside the cell, triglycerides are degraded, whereas cholesterols are liberated. Subsequently, undegraded cholesterols cluster around the arterial walls, and eventually form atherosclerotic plaques [11, 20, 21]. The mechanism of plaque formation could be the reason for a significant association between RC and MACE. A low lymphocyte and high neutrophil count are typical inflammatory responses associated with the development of atherosclerotic plaque and stent implantation [22, 23, 24]. Furthermore, RC can also induce an inflammatory response via the production of cytokines and interleukins through the plasminogen activator inhibitor-1 [25]. Taken together, RCLR is a good indicator for predicting MACE, as it considers the effects of atherosclerosis and inflammation.

Previous studies have also shown an association between a higher RC and severe cardiovascular disease. After following up more than 35,000 person-years in a cohort with ischemic heart disease, Jepsen *et al*. [26] reported that a high level of RC, but not LDL-C, was associated with an increased all-cause mortality rate. Similarly, another retrospective study from Varbo A *et al*. [27] suggested that an increase of 1 mmol/L in RC was associated with a 2.8-fold increased risk of ischemic heart disease. This increased risk was independent of the additional risk posed by the reduction in HDL-C levels. A multicenter, randomized clinical trial involving a cohort of older individuals further confirmed that a higher RC level was associated with an increased risk of MACE [28]. In addition to high RC, several studies have indicated the link between a decrease in lymphocyte count and an increase in adverse events for patients with cardiovascular disease [19, 29].

Little is known about RCLR from previous reports. Here, we described for the first time, RCLR, which combines both parameters of RC and inflammation, as a tool for the assessment of cardiovascular risk. Since most patients hospitalized for CAD were diagnosed with UA and received interventional therapy, we focused on patients with UA who underwent PCI. Our findings indicate that an elevated RCLR value was directly associated with an increased risk of MACE in patients with UA treated with PCI. Even after adjusting for confounding variables, the increased level of RCLR remained a strong indicator of all-cause death, non-fatal MI, and ischemia-driven revascularization. In addition, we performed ROC analysis to determine the risk prediction ability of RCLR. In comparison with RC, the AUCs of RCLR were larger (Fig. 3A), which suggests that RCLR had a better ability to indicate risks of non-fatal MI and MACE. However, ROC analysis did not indicate a significant difference between RCLR and RC in their ability to predict ischemia-driven revascularization. Overall, the inflammatory dimension of RCLR made it more effective than RC for risk prediction. Since lymphocyte index can be determined easily by routine laboratory examination, it should not be difficult to calculate RCLR in most circumstances. Given its comprehensive ability and capacity to predict adverse cardiovascular outcomes, RCLR may prove to be a valuable tool for guiding clinicians in treating cardiac patients.

## 5. Limitation

As this was a single-center study, the risk of bias and lack of reproducibility may affect the validity of conclusions in different conditions and diverse populations. Some baseline information was not collected, such as C-reactive protein levels and the use of lipid-lowering drugs, which may affect the accuracy of the results. We were unable to predict all-cause mortality from the ROC analysis of RCLR due to a low death rate. Additionally, we did not check whether a low RCLR leads to a reduction in the occurrence of adverse events. Thus, additional studies are needed to independently verify the results of this study.

## 6. Conclusions

A higher RCLR was directly associated with the increased incidence of MACE in patients with UA treated with PCI. In higher RCLR quartiles, all-cause death, non-fatal MI, and ischemia-driven revascularization rates increased significantly. In comparison with RC, RCLR showed a better ability to predict MACE and non-fatal MI. Collectively, RCLR appeared to be a significant tool for predicting cardiovascular risks.

## Data Availability

All data presented in this study was generated by the authors, at the affiliated institutions at the time of submission. All original raw data is available at the time of submission. This data will be available to the Editorial Office, Editors and readers upon request.

## Supplementary Material

Supplementary material associated with this article can be found, in the online version, at https://doi.org/10.31083/j.rcm2403071.
